# Brain Magnetic Resonance Imaging Radiomic Signature and Machine Learning Model Prediction of Hepatic Encephalopathy in Adult Cirrhotic Patients

**DOI:** 10.3390/life15030346

**Published:** 2025-02-22

**Authors:** Gianvincenzo Sparacia, Giulia Colelli, Giuseppe Parla, Giuseppe Mamone, Luigi Maruzzelli, Vincenzina Lo Re, Federica Avorio, Roberto Miraglia, Anna Pichiecchio

**Affiliations:** 1 Radiology Service, Department of Biomedicine, Neuroscience and Advanced Diagnosis (BiND), University of Palermo, 90127 Palermo, Italy; 2 Radiology Service, IRCCS-ISMETT, 90127 Palermo, Italygmamone@ismett.edu (G.M.); lmaruzzelli@ismett.edu (L.M.); rmiraglia@ismett.edu (R.M.); 3 NeuroLab, Computational Neuroimaging Laboratory, IRCCS-ISMETT, 90127 Palermo, Italy; 4 Advanced Imaging and Radiomic Center, IRCCS Mondino Foundation, 27100 Pavia, Italy; giulia.colelli@virgilio.it (G.C.); anna.pichiecchio@mondino.it (A.P.); 5 Neurology Service, IRCCS-ISMETT, 90127 Palermo, Italy; vlore@ismett.edu (V.L.R.); favorio@ismett.edu (F.A.); 6 Department of Brain and Behavioural Disorders, University of Pavia, 27100 Pavia, Italy

**Keywords:** hepatic encephalopathy, cirrhosis, magnetic resonance imaging, radiomics, machine learning

## Abstract

Background: Hepatic encephalopathy (HE) may arise as a possible consequence of cirrhosis. Magnetic resonance imaging (MRI) may reveal a T1-weighted hyperintensity in the globi pallidi, indicating the deposition of paramagnetic substances. The objective of this research was to implement a machine learning-based radiomic model to predict the diagnosis and severity of chronic hepatic encephalopathy in adult patients with cirrhosis. Methods: Between October 2018 and February 2020, brain magnetic resonance imaging (MRI) was conducted on adult patients, both with and without cirrhosis. The control population consisted of individuals who did not have a previous medical record of chronic liver disease. The grade of hepatic encephalopathy (HE) was determined by considering factors such as the presence of underlying liver disease, the severity of clinical symptoms, and the frequency of encephalopathic episodes. Radiomic texture analysis based on five machine learning algorithms was applied to axial T1-weighted MR images of bilateral lentiform nuclei. Using the area under the receiver operating characteristics curve, we determined the accuracy of the five machine learning-based algorithms in predicting the presence of HE and the HE grading. Results: The ultimate research cohort included 124 individuals, with 70 being cirrhotic patients and 54 being non-cirrhotic controls. Of the total number of patients, 38 had a previous occurrence of HE and, among them, 22 had a grade of HE greater than 1. The multilayer perceptron algorithm classified patients versus controls with an accuracy of 100%. The k-nearest neighbor (KNN) algorithm classified patients with or without HE with an accuracy of 76.5%. The multilayer perceptron algorithm classified HE grade (HE grade 1, HE grade ≥ 2) with an accuracy of 94.1%. Conclusions: The machine learning algorithms implemented provide a robust modeling technique for deriving valuable insights from brain MR images in cirrhotic patients and this can serve as an imaging tool valuable for the assessment of the burden of hepatic encephalopathy.

## 1. Introduction

Hepatic encephalopathy (HE) is a neurological condition that may occur in patients with advanced chronic liver disease [1]. It is marked by various neuropsychiatric symptoms. Hepatic encephalopathy (HE) is considered a significant complication of cirrhosis, since it is the first decompensating event in about 20% of these patients and is linked to a 5-year survival rate of 20% [2]. Hepatic encephalopathy (HE) is the primary reason for prolonged hospital stays and repeated admissions among patients with cirrhosis, leading to a significant economic impact [1,2]. The occurrence of hepatic encephalopathy (HE) in patients with cirrhosis over a 5-year period varies between 5% and 25%. The likelihood of developing HE depends on the specific cause of chronic liver disease and the presence of other complications, and increases to 50% in cirrhotic patients who have undergone transjugular intrahepatic portosystemic shunt (TIPS) for the management of portal hypertension complications, such as refractory ascites and the prevention of variceal rebleeding [1,2,3,4,5,6].

A frequent finding on magnetic resonance imaging (MRI) is the presence of a bilateral symmetric T1-weighted hyperintensity in the globi pallidi and substantiae nigrae. This finding is likely caused by the long-term buildup of manganese due to impaired hepatobiliary excretion and is reported in 80–90% of patients with chronic liver failure.

However, this imaging result is not directly or proportionally associated with blood ammonia levels and/or patient complaints [7], and it is only detected in chronic phases. Furthermore, it is possible to see bilateral T1-weighted hyperintensity in the globi pallidi under situations that are not connected to the buildup of manganese [8]. In cirrhotic individuals with acute encephalopathy, more complex imaging abnormalities may be noticed. This condition is often triggered by a sudden worsening of liver function, leading to a rapid increase in ammonia levels in the blood. The imaging tests of these individuals often show acute hyperammonemic encephalopathy features that are similar to the imaging findings of chronic HE. The imaging results of acute HE may show hyperintense FLAIR signals in a gyriform pattern, along with limited diffusion. These changes often impact both sides of the cerebral cortices, but usually do not include the perirolandic and occipital cortex. The occurrence of acute hyperammonemic cortical damage often overlaps with the pre-existing imaging characteristics of chronic hepatic encephalopathy, particularly the presence of hyperintense T1 signals in the globi pallidi.

Radiomics is a developing method of quantitative analysis in imaging that allows for the extraction of mathematical characteristics from radiological images that are not discernible to the human eye [9,10,11]. Radiomics and texture-based characteristics [12] provide insights on the distribution and variation of signal intensity within a specific area of interest. The collected images may be integrated into clinical decision support systems to forecast histological traits, prognosis, and therapy response. The statement elucidates the notable enthusiasm around precision medicine and the utility of this emerging approach [11]. Recent studies reported the feasibility of radiomics MRI-based texture analysis for detecting brain tumors [13,14], multiple sclerosis lesions [15,16], and other neurodegenerative illnesses [17].

Nevertheless, the influence of radiomics on routine clinical procedures in neuroradiology has been very limited so far. The primary constraint impeding clinical use is the origin of radiomic artificial intelligence (AI) and machine learning (ML) algorithms, which were first created outside the medical domain [18]. The application of these algorithms in the radiological domain has not yet achieved complete success, primarily because the existing infrastructure of radiological departments needs to be modified to accommodate the integration of AI and ML algorithms. Additionally, the absence of standardized machine learning procedures, particularly in the non-cancer neuroradiology field, further hinders their implementation [11].

In our previous study [19], the presence of hepatic encephalopathy was assessed in adult patients with liver cirrhosis using radiomic texture analysis of brain magnetic resonance imaging. In this study, the radiomic methodology is founded on statistical analysis. To realize the full potential of this analysis, it is essential to strictly adhere to key statistical principles; therefore, advanced statistical skills and knowledge are required to relate the large-scale texture dataset to clinical endpoints. This methodology has the disadvantage of being a time-consuming procedure that requires advanced statistical analysis skills for the dimensionality reduction of features and the development of a predictive model. These factors limit the clinical implementation of this method in a routine setting. In recent years, machine learning algorithms have demonstrated their enormous potential for image segmentation, reconstruction, recognition, and classification [20]. The aim of this study is to effectively implement into the clinical–neuroradiological workflow a machine learning-based radiomic algorithm for advanced model building of radiomics-based signatures or classifiers aimed at predicting hepatic encephalopathy in adult cirrhotic patients using brain magnetic resonance imaging.

## 2. Materials and Methods

This retrospective research was approved by the Institutional Research Board of our institution (approval number: 32/17). The need for informed consent was waived. All participants receiving brain tests for clinical reasons provided written informed permission for a magnetic resonance investigation (MR).

### 2.1. Patient Population

The retrospective identification of eligible patients was performed by searching the computerized database of our tertiary transplant center. This search especially focused on adult patients who had received magnetic resonance (MR) brain exams from October 2018 to February 2020. All patients were scanned using the same imaging methodology and MR equipment as detailed in prior research [19].

The research cohort included individuals diagnosed with cirrhosis who were selected based on the predetermined inclusion criteria: (1) the diagnosis of cirrhosis by diagnostic techniques; (2) the application of a 3T MR scanner for conducting brain MRI using the same scanning methodology; and (3) the analysis of pertinent clinical data to assess the existence of chronic hepatic encephalopathy.

The research cohort excluded patients who had the following criteria: (1) the presence of motion artifacts seen in axial T1-weighted images; and (2) the existence of a transjugular intrahepatic portosystemic shunt (TIPS).

The control group included individuals without chronic liver disease or chronic hepatic encephalopathy who underwent brain MRI using the same MR unit and imaging methods.

The diagnosis of cirrhosis is usually confirmed by liver biopsy or by assessing the clinical, biochemical, and morphological features of the liver using computed tomography (CT) and/or magnetic resonance imaging. The classification of hepatic encephalopathy’s clinical condition was determined by evaluating the frequency and severity of encephalopathic episodes, as well as the underlying cause of liver sickness [21]. The severity and the frequency of hepatic encephalopathy was assessed using the West Haven criteria (WHC) [1]. Hepatic encephalopathy was classified according to its time course in episodic HE, recurrent HE (which denotes bouts of HE that occur with a time interval of 6 months or less), and persistent HE (which denotes a pattern of behavioral alterations that are always present and interspersed with relapses of overt HE).

### 2.2. MR Imaging Acquisition

The MR exams were obtained using a 3T MR scanner (Discovery 750w, General Electric Healthcare, Milwaukee, WI, USA) equipped with a 32-channel head coil, following the methodology outlined in a previous paper [19]. Non-enhanced and contrast-enhanced magnetic resonance (MR) images were obtained using a fast-spin echo (FSE) T1-weighted sequence. The contrast agent used was gadobutrol (Gadovist, Bayer, Germany) at a dose of 0.1 mmol/kg. The imaging parameters included a field-of-view (FOV) of 240 mm, a matrix size of 320 × 320, and a slice thickness of 4 mm with a 1 mm intersection gap; and a number of excitations of 1. Axial and sagittal FSE T2-weighted images were also acquired using a repetition time (TR) of 8000 ms and an echo time (TE) of 90 ms. Additionally, axial fluid attenuated inversion-recovery (FLAIR) images were obtained with a TR of 9000 ms, a TE of 150 ms, and an inversion time (TI) of 2250 ms. These imaging sequences are part of the standard MR protocol.

### 2.3. Image Processing and Extraction of Texture Features

An experienced neuroradiologist, specialized in neuroimaging for 20 years, examined MRI scans to classify cirrhotic patients more precisely by identifying hyperintense signals in the basal ganglia on non-contrast T1-weighted images.

Each patient’s anonymised T1-weighted axial images were transferred in DICOM format to a workstation for the purpose of segmenting the lentiform nuclei and extracting textural information. A radiologist who has specialized in neuroimaging for six years outlined a region of interest (ROI) surrounding both sides of the lentiform nuclei in a single-slice axial T1-weighted image at the level of the Monro foramina. The radiologist was not aware of any patient clinical data throughout this process. The zone of interest was placed inside the boundaries of the lentiform nuclei and covered the largest feasible area while avoiding calcification. All participants showed no anatomical abnormalities in the basal ganglia on MRI, except for accidental mineralization.

The images were analyzed using the software package LIFEx (version 5.10, https://www.lifexsoft.org accessed on 29 January 2024). The analysis was done in a random order, and the software automatically extracted a total of 43 texture features, as described previously [19]. These features included first-order features, which were calculated by analyzing the gray-scale distribution within a defined region of interest (ROI), without taking into account the spatial relationships between pixels. Additionally, second-order features were calculated, considering the spatial relationships between pixels.

The first-order parameters consisted of traditional metrics such as the mean, standard deviation, and histogram (which represents the distribution of pixel intensities). The second-order features included the gray-level co-occurrence matrix (GLCM), which analyzed the patterns of pixel pairs to calculate texture indices; the gray-level run length matrix (GLRLM), which measured consecutive pixels with the same intensity in specific directions; the neighborhood gray-level difference matrix (NGLDM), which quantified the difference between a pixel and its eight neighboring pixels; and the gray-level zone length matrix (GLZLM). The LifeX online manual [22] offers a detailed analysis of the algorithms that control each texture element. The data used for this research are identical to the ones utilized in a previously performed examination at our institution [19].

### 2.4. Machine Learning Analysis

The dataset utilized in this analysis comprises radiomics features that have been extracted, as detailed in our prior investigation [19]. These features encompass first-order characteristics, which pertain to the intensity distributions of voxels without considering spatial pixel relationships (e.g., mean, standard deviation, and other indices of the grayscale histogram). Additionally, second-order features are included, which highlight the spatial relationships between voxels (e.g., GLCM, GLRLM, NGLDM, and GLZLM matrices). Lastly, semantic features are incorporated, encompassing clinical information and MR findings. Specifically, the latter category encompasses the MR finding “hyperintense T1”, which is utilized for the purpose of categorizing patients with cirrhosis based on the evidence of hyperintensity in the basal ganglia on non-contrast T1-weighted images, the presence of hepatic encephalopathy (HE), and the severity of HE. The dataset was subjected to scaling to account for variations in units of measurement among the features. This was done to prevent discrepancies in the feature range from impacting the effectiveness of the dimensionality reduction process.

On the resulting dataset, dimensionality reduction was implemented using principal component analysis (PCA) [23], with the aim of lowering the dimensionality of the data while preserving as much as possible data variation. The resulting PCA principal components are new latent variables obtained as linear combinations of the original features. A set of principal components that retain over 90% of the explained variance was identified and each data point was projected onto it.

Therefore, the PCA feature extraction reflects the original feature components of the sample. Since a PCA component can be expressed as a linear combination of the original features, the coefficient for each feature, or “loading”, can be used to determine the extent to which that feature contributed to the feature extraction result. The lower the contribution of a feature, the lower the absolute contribution of the associated coefficient in the PCA component. When a feature is less essential for feature extraction, we can assume that this feature component is also less important in the original space [24].

Given that there are multiple eigenvectors, it is crucial to consider more than one eigenvector when assessing the significance of a feature component. The algorithm considered for feature selection calculates the contribution of a specific component to the feature extraction results as follows [24]:

$$c_{j}=\sum_{p=1}^{m}\left|V_{pj}\right|$$

with j=1,…,N with N (the dimensionality of the initial dataset), p=1,…,m with m the number of eigenvectors and ${|V}_{pj}|$ the absolute value of the *j*-th enty of the *p*-th eigenvector $V_{p}$ [24].

The low-dimensional dataset was used as input for training machine learning algorithms after being randomly assigned to a training set (80%) and a test set (20%). The machine learning algorithms implemented included multilayer perceptron (MLP) [25], a feed-forward neural network with 100 neurons in the hidden layer, reLU activation function (rectified linear unit function), constant learning rate of 0.001, Adam solver for weight optimization, tree [26], random forest (RF) [27], k-nearest neighbor (KNN) [28], and support vector machine (SVC) [29]. These machine learning algorithms were implemented using the scikit-learn Python library version 3.11.11 [30,31].

**Figure 1:**
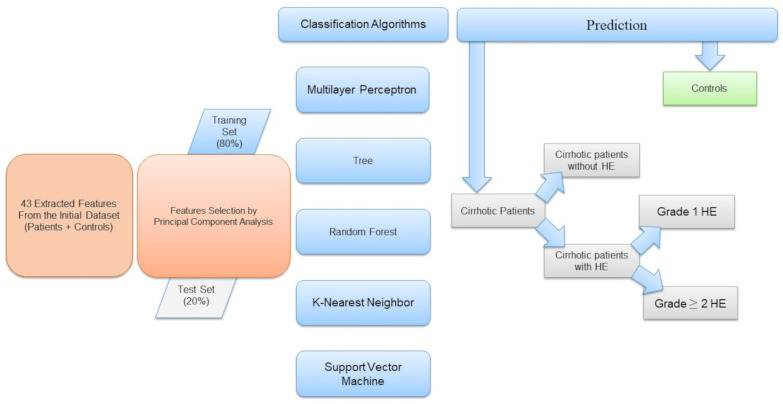
Schematic representation of the machine learning-based radiomic workflow classification of: controls and cirrhotic patients; cirrhotic patients with hepatic encephalopathy and cirrhotic patients without hepatic encephalopathy; cirrhotic patients with grade 1 hepatic encephalopathy and grade ≥ 2 hepatic encephalopathy.

Specifically, we classified subjects as patients with cirrhosis or controls. Then we selected patient data points from the dataset and randomly assigned them into a training group (80%) and a test group (20%). We classified patients according to the presence or absence of HE, then identified the HE grade (HE = 1 and HE ≥ 2). Figure 1 shows a schematic representation of the workflow.

To evaluate the performance of the implemented ML classification algorithms, we computed the corresponding confusion matrix that allows visualization of the performance of an algorithm with the misclassified elements on the outer diagonals, the receiver operating characteristic (ROC) curve, and the accuracy.

## 3. Results

### 3.1. Patients’ Characteristics

**Figure 2:**
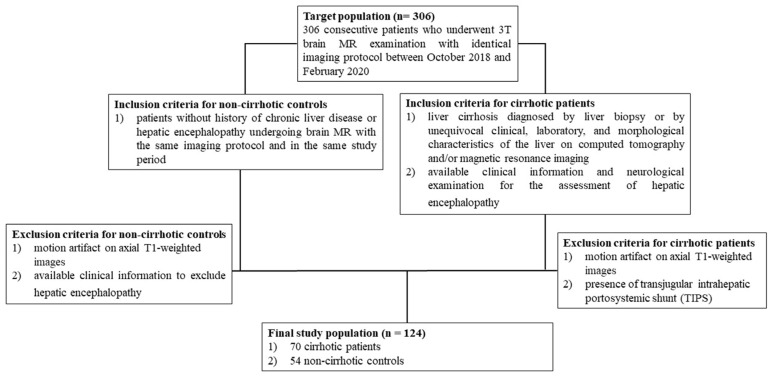
The flowchart illustrates the enrollment process of 306 consecutive patients based on specific inclusion and exclusion criteria. The figure is sourced from a previously published study [19].

The research sample consisted of 124 participants, with 70 patients with cirrhosis (74% male, mean age 66 ± 8 years, range 40 to 86 years) and 54 noncirrhotic control patients (69% male, mean age 62 ± 13 years, range 28 to 81 years). Figure 2 illustrates the criteria used to determine which patients were included or excluded from the study’s population and control group, as outlined in a prior investigation [19].

The baseline data of the patients involved in the investigation are shown in Table 1, as documented in the previous study [19].

The clinical reasons for doing brain MRI in cirrhotic patients were as follows: pre-transplantation evaluations in 52 patients (74%); assessment of signal intensity changes in the globi pallidi associated with chronic HE in 8 patients (12%); and evaluation of additional neurological symptoms in 10 patients (14%). Chronic hepatic encephalopathy (HE) was diagnosed in 38 of the cirrhotic individuals, accounting for 54% of the total. Grade 1 HE was seen in 16 patients, which accounted for 23% of the total. On the other hand, 22 patients, or 32% of the total, had HE grade ≥ 2. Episodic, recurring, and persistent hepatic encephalopathy (HE) were seen in 14 (20%), 11 (16%), and 13 (19%) individuals, respectively. Brain MRI was clinically indicated in patients without a history of chronic liver disease or hepatic encephalopathy for various reasons. These included memory impairment in 6 (11%) patients, migraine in 11 (20%) patients, chronic hypoxic-ischemic encephalopathy in 31 (57%) patients, optic neuropathy in 2 (4%) patients, and cortical laminar necrosis in 4 (8%) patients.

### 3.2. Classification Patients Versus Controls

**Figure 3:**
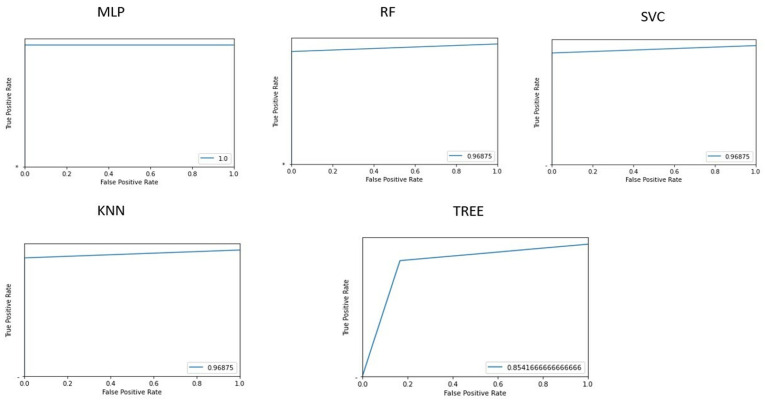
Graphs representing the AUROC in classifying patients and controls for each machine learning algorithm implemented. AUROC = area under the receiver operating characteristic curve; MLP = Multilayer perceptron; RF = Random Forest; SVC = Support vector machine; KNN = k-nearest neighbor.

Figure 3 shows the area under the receiver operating characteristic curves for the different implemented machine learning algorithms.

**Figure 4:**
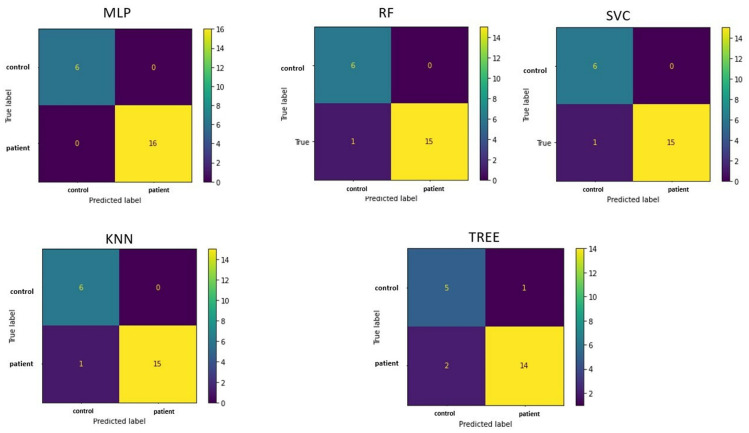
Confusion matrix in classifying patients and controls for each machine learning algorithm implemented. MLP = Multilayer perceptron; RF = Random Forest; SVC = Support vector machine; KNN = k-nearest neighbor.

The confusion matrices in Figure 4 allow visualization of the performance of the ML algorithms.

Table 2 shows the accuracy of classifying patients and controls for each implemented machine learning algorithm, along with values. The multilayer perceptron algorithm demonstrated the best accuracy (100%).

Based on feature selection from the implemented PCA, the 10 most influential features for the classification task were: GLRLM_SRHGE, HISTO_Skewness, GLRLM_GLNU, HISTO_Kurtosis, HISTO_ExcessKurtosis, NGLDM_Contrast, HYPERINTENSE T1, GLZLM_SZHGE, NGLDM_Coarseness, and NGLDM_Busyness.

### 3.3. Classification of Patients with or Without HE

**Figure 5:**
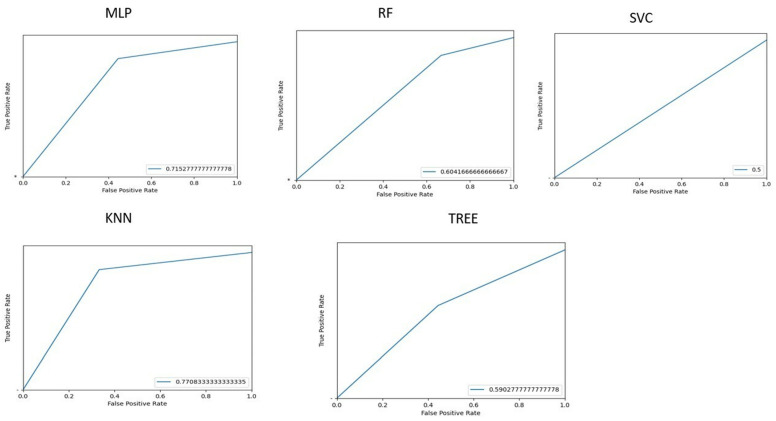
Graphs representing AUROC in classifying cirrhotic patients with hepatic encephalopathy and cirrhotic patients without hepatic encephalopathy for each implemented machine learning algorithm. AUROC = area under the receiver operating characteristic curve; MLP = Multilayer perceptron; RF = Random Forest; KNN = k-nearest neighbor; SVC = Support vector machine; HE = hepatic encephalopathy.

To identify patients with HE, we implemented ML algorithms, and the corresponding ROC curves and AUC values are shown in Figure 5.

**Figure 6:**
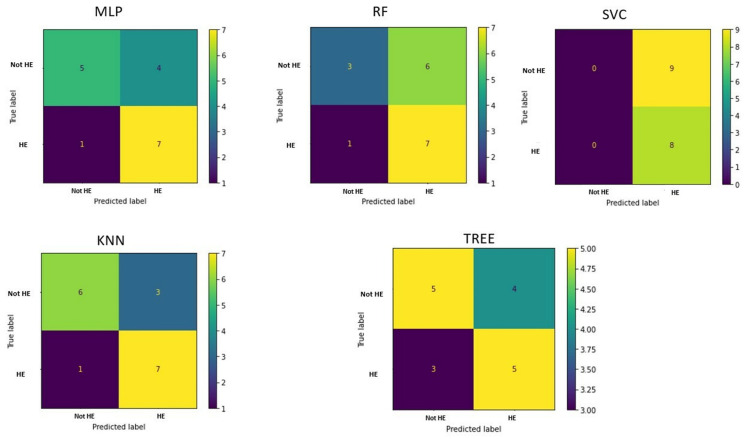
Confusion matrix for classifying cirrhotic patients with hepatic encephalopathy (HE) and cirrhotic patients without HE for each implemented machine learning algorithm. MLP = Multilayer perceptron; RF = Random Forest; SVC = Support vector machine; KNN = k-nearest neighbor.

The confusion matrices are shown in Figure 6.

Table 3 shows the accuracy of each machine learning algorithms in classifying cirrhotic patients with or without hepatic encephalopathy.

It can be deduced that KNN gives the best prediction performance, with an accuracy of 76.5%. Considering the linear combination of PCA components, the most significant features were HISTO_Kurtosis, Indication for examination, NGLDM_Contrast, GLZLM_SZHGE, NGLDM_Busyness, GLRLM_GLNU, NGLDM_Coarseness, and HYPERINTENSE T1.

### 3.4. Classification of HE Grade (HE Grade 1, HE Grade ≥ 2)

**Figure 7:**
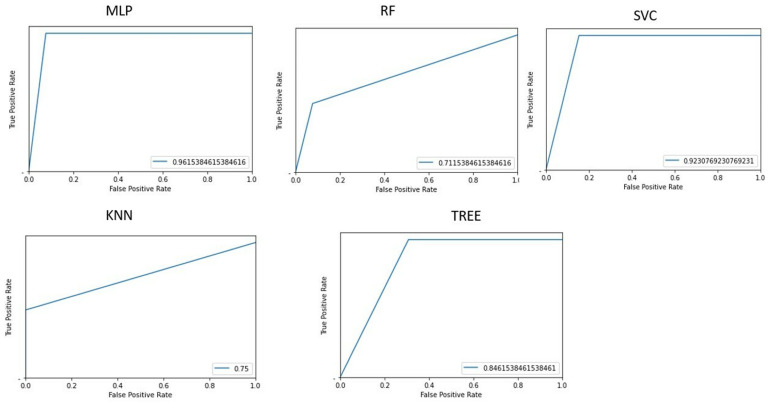
Graphs representing the area under the receiver operating characteristic curve (AUROC) for each machine learning algorithm implemented for classification of hepatic encephalopathy (HE) patients with an HE grade = 1 or HE grade ≥ 2.

Figure 7 shows the area under the receiver operating characteristic curve for the different implemented machine learning algorithms when differentiating HE patients with an HE grade = 1 and HE grade ≥ 2.

**Figure 8:**
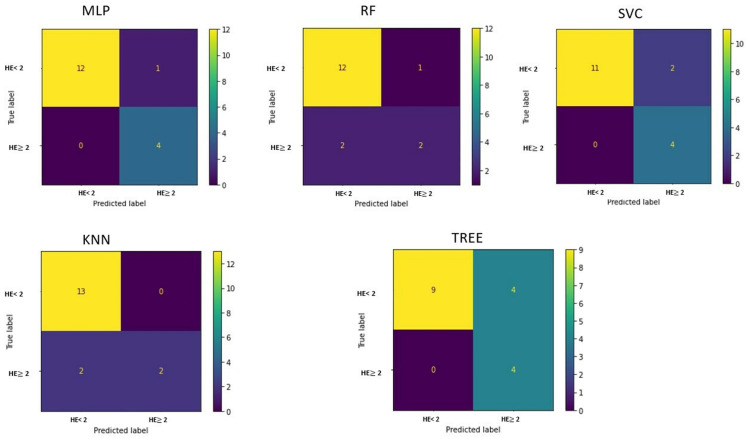
Confusion matrix for classification of hepatic encephalopathy (HE) patients with an HE grade = 1 or HE grade ≥ 2 for each implemented machine learning algorithm.

The confusion matrices are shown in Figure 8.

The best performance in differentiating patients by HE grade is obtained with the implementation of a multilayer perceptron, which has an accuracy of 94.11% (Table 4).

Using the information on PCA loadings, we can deduce that the most influential 10 features were HE, HISTO_Kurtosis, HISTO_ExcessKurtosis, NGLDM_Contrast, GLZLM_SZHGE, GLRLM_GLNU, HYPERINTENSE T1, TIPS, NGLDM_Coarseness, and Indication for examination.

## 4. Discussion

Our study shows that employing multiple machine learning algorithms for radiomics analysis is practical and can be applied in regular clinical environments to predict various clinical findings for the identification and staging of hepatic encephalopathy in patients with cirrhosis using texture features extracted from T1-weighted imaging.

Among the diverse ML algorithms used in this study, the multilayer perceptron algorithm allowed the classification of cirrhotic patients with 100% accuracy. The classification of patients with or without HE was best predicted using the KNN algorithm, which had an accuracy of 76.5%. The classification of HE grades (HE grades 1 and 2) was best predicted by using the multilayer perceptron algorithm, which had an accuracy of 100% and 76.5%, respectively.

The underlying mechanism of hepatic encephalopathy in cirrhosis is not well understood. In the presence of substantial liver failure, the liver becomes more resistant to the usual flow of substances between the liver and intestines. This causes toxins, such as ammonia and inflammatory mediators, that originate in the gut to enter the bloodstream via spontaneous connections between the portal and systemic circulations. The brain subsequently absorbs ammonia, which has been associated with edematous changes that impact astrocytes and neurons [5]. Prolonged exposure to cerebral ammonia leads to structural changes in astrocytes. In addition to inflammation, oxidative stress, an elevation in bile acids, and lactate all have a role in the progression and intensity of HE [6].

HE was classified using the West Haven Criteria (WHC) and the International Society for Hepatic Encephalopathy and Nitrogen Metabolism (ISHEN) [1]. The ISHEN distinguishes between concealed and open hazardous events. Patients with covert hepatic encephalopathy (HE) have little or no noticeable clinical symptoms and usually do not need to be admitted to the hospital. On the other hand, patients with overt HE experience confusion in terms of time and space or show signs of asterixis, and they generally need to be hospitalized [3]. The WHC categorization consists of six stages: unimpaired, minimum, and categories I through IV of HE. From a clinical standpoint, it is difficult to differentiate between minor hepatic encephalopathy (HE) and grade I HE. However, grades II through IV indicate obvious HE and vary in intensity, ranging from asterixis in grade II to coma in grade IV [1].

Our hypothesis suggests that analyzing the radiomics of globi pallidi might effectively capture the variation in different brain areas affected by cirrhosis and hepatic encephalopathy. This analysis has the potential to accurately identify these conditions in patients.

The radiomics-based method may be used to enhance the accuracy of MRI diagnosis of hepatic encephalopathy (HE), in particular, in cirrhotic patients who are having MR imaging for monitoring and have minor neurological symptoms. These findings can be further validated by clinical examination. Utilizing brain MR radiomic features to differentiate between cirrhotic and non-cirrhotic individuals might assist in identifying the root cause of chronic liver disease in patients who are having brain MRI for unrelated purposes.

To enhance patient stratification before the TIPS treatment, our study’s first findings may stimulate further investigation, such as the exploration of radiomic features that might potentially predict post-TIPS HE. The most notable drawback of TIPS is the occurrence of post-TIPS hepatic encephalopathy, which has been documented to affect 30–50% of patients. Although symptoms are generally mild in most patients, they may be severe in some cases, leading to the need for hospitalization. After the TIPS procedure, the occurrence of post-TIPS hepatic encephalopathy (HE) puts a significant financial strain on healthcare systems. A recent study involving around 28,000 hospitalized patients who underwent the TIPS procedure found that 28% of them were readmitted within 30 days. Among these readmissions, HE, with or without coma, was the primary reason for at least one-third of the patients [17]. Various clinical variables, including advanced age, a previous occurrence of hepatic encephalopathy (HE), a higher Child-Pugh/MELD score, compromised nutritional status, and low levels of blood sodium, have been linked to the development of post-Tips hepatic encephalopathy [16]. However, precisely identifying individuals at risk before undergoing TIPS remains challenging. Hepatologists are cautious about suggesting Transjugular Intrahepatic Portosystemic Shunt (TIPS), owing to the potential for post-TIPS Hepatic Encephalopathy (HE), especially in patients with persistent ascites. In these individuals, the survival rate after TIPS is lower than in patients with variceal hemorrhage as the main reason for undergoing TIPS. Incorporating a brain radiomic signature as an adjunctive method to enhance patient selection for TIPS has the potential to hypothetically reduce the occurrence of post-TIPS HE.

In this research, we evaluated a machine learning approach to analyze first-order, second-order, and semantic features extracted from the regions of interest (ROIs) encompassing the globi pallidi obtained in a previous study [19] in which statistical analysis was used to reduce the dimensionality of features and for clinical prediction. This study utilized an approach based on machine learning to prevent overfitting or inaccurate estimates of generalization and to facilitate the routine clinical implementation of radiomic analysis [32].

The results of this study, in which feature selection and extraction were based on an unsupervised method employing principal component analysis (PCA) [23], and the model prediction was based on five machine learning algorithms (MLP, Tree, RF, KNN, and SVC) [25,26,27,28,29,30,31], indicate that this method can achieve clinical prediction results comparable to those previously obtained [19], as summarized in Table 5, with the machine learning approach having the advantage of a shorter time to achieve the same results, thus facilitating the implementation in routine clinical settings.

The current research has certain limitations that need acknowledgment. This research was conducted retrospectively. The inter-reader agreement for stratifying cirrhotic patients based on the MR finding of T1-weighted hyperintensity in the basal ganglia, as well as the placement of regions of interest (ROIs) on T1-weighted images for texture extraction, was not examined in our research.

Every participant underwent imaging utilizing a 3T magnetic resonance (MR) scanner. Hence, it is crucial to note that the results of this research may not be relevant to 1.5-T MR scanners, which remain extensively used for the clinical assessment of cirrhotic patients. The mean MELD score of our cohort of cirrhotic patients was 14, indicating the existence of advanced liver disease. It is recommended that these results be reproduced in patients with less progressed liver disease.

An additional drawback of this retrospective investigation was the failure to comply with the recommendations set by the Quantitative Imaging Biomarkers Alliance (QIBA) to achieve repeatability of the radiomic characteristics. Reproducibility, as described by the QIBA, involves obtaining measurements that can be repeated in many conditions, including different locations, with different operators, or with different scanners [33]. The absence of replicability in imaging data significantly contributes to the substantial variability of radiomic biomarkers. This variability may compromise the accuracy and reliability of prediction models based on radiomics. Consequently, the capacity of these models to be used effectively on large groups of patients and patients scanned with different MR machines may be hindered [34]. In spite of the aforementioned limitations, this study suggests that machine learning algorithms offer robust modeling techniques for extracting valuable insights from brain MR images, thereby revealing complex biological mechanisms and enabling the potential for tailored precision diagnosis and treatment planning in cirrhotic patients to prevent the burden of hepatic encephalopathy.

## 5. Conclusions

In this study, the machine learning algorithm implemented provides robust modeling techniques for deriving valuable insights from brain MR images in adult cirrhotic patients with hepatic encephalopathy. The radiomic signature outperforms traditional imaging models in the diagnosis and prediction of hepatic encephalopathy in adult cirrhotic patients, and this could potentially improve clinical diagnosis and management of these patients.

## Figures and Tables

**Table 1:** Characteristics of included patients. Previously published table [19].

Characteristics	Cirrhotic (*n* = 70)	Controls (*n* = 55)
Age (years) mean ± SD (range)	66.2 ± 8.4 (40–86)	62.4 ± 13 (28–81)
Sex		
Men	52 (74%)	37 (69%)
Women	18 (26%)	17 (31%)
Etiology of the cirrhosis		
Viral	41 (59%)	-
NASH	11 (16%)	-
Alcohol	10 (14%)	-
Others	8 (11%)	-
Hepatic encephalopathy	38 (54%)	0 (0)
HE Grade		
Grade 1	16 (23%)	-
Grade 2	16 (23%)	-
Grade 3	4 (6%)	-
Grade 4	2 (3%)	-
Type of hepatic encephalopathy		
Episodic	14 (20%)	-
Recurrent	11 (16%)	-
Persistent	13 (19%)	-
MELD, mean ± SD (range)	14.0 ± 5.6 (6–40)	-

**Table 2:** Accuracy in classifying patients with cirrhosis and controls for each implemented machine learning algorithm.

Algorithm	Accuracy	AUROC
MLP	100%	1
Tree	86.36%	0.85
RF	95.5%	0.96
KNN	95.5%	0.96
SVC	95.5%	0.96

AUROC = area under the receiver operating characteristic curve; MLP = Multilayer perceptron; RF = Random Forest; KNN = k-nearest neighbor; SVC = Support vector machine.

**Table 3:** Accuracy in classifying cirrhotic patients with hepatic encephalopathy and cirrhotic patients without hepatic encephalopathy for each implemented machine learning algorithm.

Algorithm	Accuracy	AUROC
MLP	71%	0.71
Tree	58.82%	0.59
RF	58.82%	0.60
KNN	76.5%	0.77
SVC	47%	0.5

AUROC = area under the receiver operating characteristic curve; MLP = multilayer perceptron; RF = Random Forest; KNN = k-nearest neighbor; SVC = Support vector machine.

**Table 4:** Accuracy in classifying grade 1 hepatic encephalopathy and grade ≥ 2 hepatic encephalopathy in cirrhotic patients for each implemented ML algorithm.

Algorithm	Accuracy	AUROC
MLP	94.11%	0.96
Tree	76.47%	0.84
RF	82.35%	0.71
KNN	88.24%	0.75
SVC Linear	88.24%	0.92

AUROC = area under the receiver operating characteristic curve; MLP = Multilayer perceptron; RF = Random Forest; KNN = k-nearest neighbor; SVC = Support vector machine.

**Table 5:** The presented table shows the AUROC and accuracy of the radiomic predictive model for evaluating cirrhosis, hepatic encephalopathy (HE), and the grade of HE. The assessment is conducted using two different methodologies: the statistical approach employed in a prior study [19].

Outcome Prediction	Statistical Method			Machine Learning Method			
	AUROC	Accuracy	*p* Value	Best Machine Learning Algorithm for Prediction	AUROC	Accuracy	*p* Value
Cirrhosis	0.97	93%	<0.0001	MLP	1	100%	<0.0001
HE (any grade)	0.82	71%	<0.0001	KNN	0.77	76.5%	<0.0001
HE = 1 vs. HE ≥ 2	0.75	63%	<0.0001	MLP	0.96	94.1%	<0.0001

HE = Hepatic encephalopathy; AUROC = Area Under the Receiver Operator Characteristics curve; MLP = Multilayer perceptron; KNN = k-nearest neighbor.

## Data Availability

The data presented in this study are available on request from the corresponding author. The data are not publicly available due to privacy or ethical policy.
